# Pathophysiology of Rejection in Kidney Transplantation

**DOI:** 10.3390/jcm12124130

**Published:** 2023-06-19

**Authors:** Christina L. Tamargo, Sam Kant

**Affiliations:** 1 Department of Medicine, Johns Hopkins University School of Medicine, Baltimore, MD 21224, USA; ctamarg1@jh.edu; 2 Division of Nephrology & Comprehensive Transplant Center, Department of Medicine, Johns Hopkins University School of Medicine, Baltimore, MD 21224, USA

**Keywords:** allograft rejection, antibody-mediated rejection, kidney transplant, T-cell-mediated rejection, transplant immunology

## Abstract

Kidney transplantation has been the optimal treatment for end-stage kidney disease for almost 70 years, with increasing frequency over this period. Despite the prevalence of the procedure, allograft rejection continues to impact transplant recipients, with consequences ranging from hospitalization to allograft failure. Rates of rejection have declined over time, which has been largely attributed to developments in immunosuppressive therapy, understanding of the immune system, and monitoring. Developments in these therapies, as well as an improved understanding of rejection risk and the epidemiology of rejection, are dependent on a foundational understanding of the pathophysiology of rejection. This review explains the interconnected mechanisms behind antibody-mediated and T-cell-mediated rejection and highlights how these processes contribute to outcomes and can inform future progress.

## 1. Introduction

Kidney transplantation is the preferred treatment for end-stage kidney disease and is the most common type of organ transplant, with over 25,000 kidney transplants performed in the US in 2022 [1,2]. When the first successful human-to-human transplant was performed in 1954, there was no knowledge of HLA types, and the transplant was successful only because it was performed on identical twins [3,4]. In the years that followed, HLA was discovered, crossmatch testing was invented, and transplantation became a more viable therapeutic tool. Immunosuppressive medications started to be used in rejection episodes in the 1970s, but induction therapy and maintenance immunosuppression did not become widespread until the 1980s and 1990s. In recent decades, the field of transplants has continued to evolve in terms of research, knowledge, tools, and implementation.

Despite the increased prevalence of and familiarity with transplantation and therapeutic advances, allograft rejection continues to cause morbidity and mortality among transplant recipients. To evaluate the challenges at hand, the improvements that have been made, and avenues for further progress, it is imperative to understand mechanisms that underlie renal allograft rejection. This review discusses the risk of rejection, the epidemiology of rejection, the mechanisms and pathophysiology underlying antibody-mediated and T-cell-mediated rejection (ABMR and TCMR, respectively), and recent progress that has been made in understanding rejection.

## 2. Evaluation of the Risk of Rejection

Potential donors and recipients undergo an evaluation to determine the risk of rejection if transplantation occurs. Risks include inherent immunologic factors such as human leukocyte antigen (HLA) types, as well as demographic and other factors less clearly linked to immunogenicity. The former can be divided into two major risk factors: increased HLA disparity between the recipient and the donor and increased preformed donor-specific antibodies (DSA) [5].

### 2.1. HLA Typing

HLA, also known as major histocompatibility complex (MHC) molecules, are molecules expressed on cell surfaces that help the immune system distinguish between self and foreign cells. They present peptides to T cells, which allows the immune system to eliminate what is seen as foreign and to recognize the self as non-foreign. In transplantation, HLA molecules from the donor are recognized by the recipient’s immune system, and HLA molecules, therefore, serve as the major molecules implicated in graft rejection. There are three HLA regions: classes I, II, and III. Classes I and II contain the HLA genes that play a role in immunogenicity. Class I MHC molecules are composed of an alpha chain bound to beta-2 microglobulin and include the highly polymorphic HLA-A, HLA-B, and HLA-C, as well as the nonclassical and less polymorphic molecules: HLA-E, HLA-F, and HLA g [6]. Class II MHC molecules are composed of an alpha chain and a beta chain and include the classical molecules HLA-DR, HLA-DQ, HLA-DP, HLA-DM, and HLA-DO, among others. CD8 T cells bind to class I molecules, while CD4 T cells bind to class II molecules. The class III region of HLA contains genes that contribute to inflammatory responses and complement activation. HLA genes are encoded close to each other on chromosome 6 and are therefore inherited in groups, or haplotypes, with one haplotype from each parent. As many HLA genes are highly polymorphic, close relatives are most likely to have the same haplotype [7].

HLA mismatch has long been implicated in the risk of allograft failure, and multiple studies, including a recent study of 189,141 first adult kidney alone transplants in the United Network for Organ Sharing (UNOS) database, support the concept that a greater number of HLA mismatches is associated with worsening allograft survival [8,9]. There is controversy surrounding the importance of HLA matching, with some suggesting that HLA matching is decreasing in significance [10], but the evidence still supports improved long-term graft survival with better HLA matching. HLA matching has been associated with other favorable outcomes as well, including a lower risk of acute rejection [11,12,13,14], a lower risk of death with a functioning graft [15], and a lower risk of post-transplant lymphoproliferative disorder [16].

Given the implications of HLA matching, HLA typing is now a critical component of the transplantation process. Typing of donors for kidney donation has traditionally focused on *HLA-A*, *HLA-B,* and *HLA-DR* loci. Several studies have shown that matching of certain HLA antigens differentially impacts outcomes relative to other antigens, and thus typing has focused on some loci more than others. For example, *HLA-B* and *HLA-DR* matching are strongly associated with patient and graft outcomes, perhaps more so than with *HLA-A* or less widely recognized antigens such as HLA-DP [14,17,18]. However, with advances in technology and a shift from serologic assays to DNA-based techniques, we are now able to type all loci more accurately. The United Network of Organ Sharing (UNOS) now requires typing of all loci in deceased donor transplants using molecular methods. *HLA-A*, *HLA-B*, and *HLA-DR* loci remain the focus for organ allocation in many centers, but there is wide variation in this; for example, some centers routinely assess other classical genes such as *HLA-DP* and *HLA-C*. HLA-DQA and HLA-DPB typing for deceased donors are conducted to evaluate for the presence of preformed antibodies in a potential recipient but otherwise is less common.

In addition to institutional variation in antibody determination, detection methods, and tools are also varied. Over 35,000 HLA class I and II alleles and 20,000 HLA proteins have been identified, making allelic typing quite burdensome, but this can be performed via high-resolution sequencing methods. Only low-resolution typing, which evaluates at the antigen rather than allelic level, is required for solid organ transplantation, so low-resolution methods are more commonly used [19,20]. These methods include sequence-specific primer (SSP) and sequence-specific oligonucleotide (SSO) technologies and variations thereof, including reverse-SSO [21]. Many commercially available techniques use Luminex-based technologies for both typing and antibody identification, and the nuances of these technologies in antibody identification are discussed in greater detail in the following sections.

### 2.2. Donor-Specific Antibodies

The presence of preformed antibodies against HLA, known as preformed donor-specific antibodies (DSA), increases the risk of transplant rejection and is associated with decreased graft survival [22,23]. Humans can develop such antibodies through exposure to foreign antigens such as previous transplants, blood transfusions, and pregnancies. Patients are screened for DSA prior to transplant, but the interpretation and implications of screening results are complex. The risk of transplant rejection may vary based on the etiology of the preformed antibody; for example, one study found that antibodies induced by pregnancy had a significantly increased rise from pre-transplant to peak DSA compared to those sensitized by previous transplants, which could perhaps have implications in risk assessment prior to transplant [24]. The degree to which patients are restricted from organs against which they have DSA is controversial and varies based on detection technique. DSA detected using complement-dependent cytotoxicity (CDC) crossmatch is typically considered a factor for not pursuingcon transplantation, because of the high risk of early rejection and graft loss [23,25], but solid-phase techniques such as enzyme-linked immunosorbent assay and Luminex-based assays have higher sensitivity for detecting DSA with unclear impact on graft outcome [26,27,28]. Positive flow cytometry, another crossmatch mechanism such as CDC, likely confers some intermediate risk of rejection and graft failure [29,30].

While Luminex-based assays have become more widely used and confer this benefit of increased sensitivity, results can vary based on the kit and laboratory used [31]. This is partly because the output of Luminex-based assays is the mean fluorescence intensity (MFI) value, which is semi-quantitative and for which each laboratory defines its own MFI cutoff [31,32]. This value can be adjusted based on a variety of features, including the HLA target and patient factors. Furthermore, even with a given antibody and MFI cutoff, a prozone effect can sometimes be observed in which low MFI values can be associated with high titer antibodies; laboratories have found ways to counteract this, though this is still imperfect as multiple mechanisms may contribute to this phenomenon [31,33]. On the other hand, some have identified methods that can increase the predictive ability of DSA, such as assessing their complement-binding activity. In a prospective cohort of 139 patients with ABMR, for example, C1q-binding anti-HLA DSA was identified as an independent determinant for allograft loss [34]. A larger study of over one thousand patients found that patients with complement-binding anti-HLA DSA after transplantation had a five-year rate of graft survival of 54%, compared to those with non-complement-binding DSA (93%) and those without any DSA (94%), as well as significantly higher rates of graft loss and ABMR.

In addition to preformed DSA that exists prior to transplantation, there is a type of DSA, as indicated by these two studies, that develops or is detected only after transplantation, and has also been identified as a risk factor for ABMR and graft failure [35,36,37,38,39]. Recent studies have examined the epidemiology of and risk factors for developing de novo DSA (dnDSA). In one prospective study of 508 kidney transplant recipients without preformed DSA who were deemed low-risk, 64 (12.6%) developed dnDSA, with nonadherence to pharmacologic therapy being a major risk factor [36]. In this study, delayed graft function, mean MFI sum score, tubulitis or chronic glomerulopathy on biopsy, and nonadherence were independent risk factors for post-dnDSA graft survival. In another study of 315 patients without preformed DSA, 47 (15%) developed dnDSA, and nonadherence was again recognized as a major risk factor, as well as HLA-DRB1 mismatches; nonadherence, delayed graft function, younger age, rejection episodes prior to dsDNA, and dnDSA were independent predictors for graft loss [37]. A prospective study that evaluated patients across a wide range of immunological risks (defined by ABO incompatibility, high panel reactive antibodies or PRA, and high pretransplant DSA MFI), including some with preformed DSA, found that 16% of patients developed dnDSA by three months and 23% by 12 months, with higher rates at 12 months in the higher-risk groups; dnDSA was again associated with a higher risk of ABMR [35].

Thus, preformed and de novo DSA play meaningful roles in transplant immunology and are becoming more prominent diagnostically and prognostically. Assessment and interpretation standards for DSA are not currently established, but efforts are being made in this realm, as evidenced by the consensus report by Lefaucher et al. and the Sensitization in Transplantation: Assessment of Risk 2022 working group [40].

### 2.3. Other Factors

In addition to HLA mismatching and the presence of a DSA, blood group incompatibility, and high PRA, a broad indicator of sensitization and immune reactivity of a potential transplant recipient, increase the risk of rejection [41]. Other less obvious immunologic factors that increase the risk include younger recipient age, older donor age, African American ethnicity if in the US, and delayed onset of graft function. Genetic predispositions have been implicated, but more research is needed to determine their roles in transplant outcomes.

With regard to these additional risk factors, younger recipients may have worse outcomes because of altered metabolism of immunosuppressive drugs, which changes in the shift from childhood to adulthood, as well as nonadherence to these medications, and others have proposed additional mechanisms such as expanded alloreactivity due to exposure to viral infections throughout childhood [42,43,44,45]. The increased risk with older donors has been demonstrated time and again with a variety of proposed mechanisms; some postulate this is due to decreased kidney function with older age, but others have shown it may instead be more closely linked to rapid loss of kidney function after the first year posttransplant [46,47,48]. Discrepancies due to race also have many causes, including a lower rate of living donor kidney transplantations in black patients compared to white patients, which has been linked to socioeconomic conditions impairing access to and knowledge of the transplant process; racism in the healthcare system; and systemic structural barriers such as the historical use of race-based glomerular filtration rate [49]. Some have also proposed that as populations with African ancestry have more HLA polymorphisms than other populations, HLA matching may be suboptimal in black populations [50,51]. Delayed graft function may be associated with a greater risk of rejection because it often requires a reduction in nephrotoxic immunosuppressive agents and because, as with much kidney injury in native kidneys, delayed graft function causes immune activation that can cause rejection downstream [52].

Finally, genetic predispositions have been implicated in the risk factor matrix, but as of yet, most associations have been weak, not reproducible, or both [53]. One polymorphism of interest that has been more heavily researched is the IIIA polymorphism in the functional Fc gamma receptor, which is on a variety of different immune cells, such as natural killer cells and macrophages. Previous studies have found histologic changes associated with ABMR, greater interferon-gamma production, and a greater degree of immune cell activation in patients or experiments involving certain polymorphisms, but these have not mapped to outcomes or survival differences in transplant [54,55]. This is a relatively under-researched area in transplant, and a greater quantity and power of studies with an increased ability to assess for genetic predispositions may eventually demonstrate risk associations that have not yet been identified.

## 3. Mechanisms for Priming T Cells

There are two forms of immune response to foreign pathogens: innate and adaptive immunity. In innate immunity, macrophages, neutrophils, natural killer cells, cytokines, acute-phase reactants, and complement provide nonspecific immune responses and do not generate any lasting memory of the pathogen [56]. In adaptive immunity, the immune system recognizes specific foreign pathogens and confers memory of such pathogens that result in robust immune responses on repeat exposure to the same pathogens. As MHC molecules on donor cell surfaces are the primary targets of the immune system in kidney transplants, adaptive immunity is the main driver in allograft rejection (though an emerging role of innate allorecognition by monocytes and natural killer cells has recently been identified [57]). B and T cells are primarily responsible for the development of adaptive immunity, with T cell priming beginning this process.

**Figure 1:**
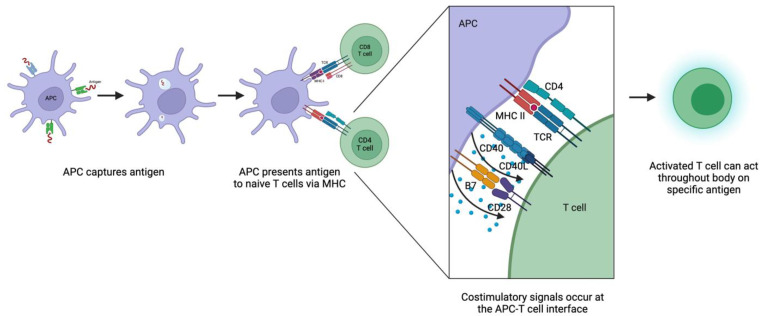
Mechanisms of T cell priming and activation. APC, antigen-presenting cell; MHC, major histocompatibility complex; TCR, T cell receptor. Created with Biorender.

Priming refers to the initial encounter between a naïve T cell and an antigen, and the activation and clonal expansion that results [58] (Figure 1). Multiple mechanisms underlie priming. First, adaptive immune responses start not at the site of the initial pathogenic insult but rather in peripheral lymphoid tissues that naïve T cells frequent. Naïve T cells enter lymphoid tissues by crossing high endothelial venules and circulate continuously between blood and lymphoid organs. Pathogens are taken to lymphoid tissues via lymph or blood, often by dendritic cells. Notably, while T cells are part of the adaptive immune system, the two immune systems are closely intertwined, and the innate immune system plays roles in T cell priming via inflammatory responses, production of substances that guide cell migration, and other means.

The next crucial part of T cell priming is the exposure of T cells to antigens. Recipient (self) antigen-presenting cells (APCs) in lymphoid tissues, such as dendritic cells and macrophages, capture antigens and present them to T cells via surface MHC molecules [59]. B cells in lymphoid tissues can also take up soluble antigens when they bind to B cell surface immunoglobulin and later present such antigens to T cells via MHC. As T cells circulate between blood and lymph, they are exposed to these MHC-peptide complexes, which reinforces positive selection for self-MHC recognition and allows one person’s pool of T cells to respond to a wide variety of antigens. T cells that do not encounter specific antigens leave the lymphoid tissue, but T cells that recognize specific antigen stop migrating and start the process of becoming effector cells. T cell migration depends on cell adhesion molecules such as selectins and integrins, some of which also play a role in T cell interactions with APCs.

Various adhesion molecules facilitate the binding between T cells and APCs. On their initial encounter, for example, CD2 on the naïve T cell binds to CD58 on the APC, among other interactions. This allows the T cell to sample many MHC molecules on APCs for the presence of specific peptides. If the T cell recognizes its specific peptide, conformational changes occur between adhesion molecules that then stabilize the bonds and allow time for the T cell to differentiate into effector T cells. The T cell that does not recognize antigens, the more common outcome, dissociates from the APC.

For the T cell to proliferate and differentiate into effector T cells, it must interact not only with the MHC-peptide complex but also with a CD4 or CD8 co-receptor, and an additional costimulatory signal is required. CD4 is a T cell surface protein that binds to the MHC class II molecule and CD8 to the MHC class I molecule. During antigen recognition, CD4 or CD8 molecules, depending on the type of T cell, associate with the T cell receptor and the MHC component of the MHC-peptide ligand [60]. The main costimulatory molecules on APCs are B7 molecules, which bind to the T cell protein CD28; these interactions provide the signals for the clonal expansion of naïve T cells. Once activated, the T cell then expresses a variety of proteins that maintain or adjust the costimulatory signal and promote clonal expansion and differentiation. Examples include the CD40 ligand, which binds to CD40 on APCs, and CD28-related proteins, such as CTLA-4, which binds to B7 on APCs. APCs express B7 molecules by way of the innate immune system, demonstrating again the interconnectedness of innate and adaptive immunity.

This form of allorecognition, mediated by self-APCs, is called the indirect pathway of allorecognition. The direct pathway, which is mediated by donor APCs, similarly produces specific T cell clones but tends to cause rejection earlier on. In this pathway, donor MHC presents peptides from endogenous proteins to T cells to start the priming process [61]. This pathway is thought to be so meaningful early on because of the frequent contact between T cells and donor cells shortly after transplant [62]. Notably, this pathway has only been demonstrated in the context of alloimmunity. Another pathway, called the semidirect pathway, adds a layer of complexity to this simplistic dichotomy and describes a mechanism of cross-regulation in which recipient dendritic cells acquire donor MHC molecules and thereby sensitize T cells via direct and indirect pathways [63].

Once an effector clone has been created by these mechanisms, progeny can act on cells throughout the body that displays the clone’s specific antigen. Effector T cells can serve a variety of functions, including killing infected cells (CD8 cytotoxic T cells), activating macrophages (TH1 cells, a type of CD4 helper T cell), and activating B cells (TH1 and TH2 cells). There is another subset of T cells, regulatory T (Treg) cells, and their fates as Tregs are typically determined far upstream in the thymus; these cells help induce and maintain immune tolerance and have crucial roles in autoimmunity and transplant [64,65]

## 4. Mechanisms of Antibody Formation

B cell activity and antibody production are closely related and s similar to T cell activation. B cells, however, undergo a more extensive maturation process before reaching secondary lymphoid organs, and the maturation process is uniquely accompanied by immunoglobulin gene rearrangements [66]. An antibody is composed of two identical light chains and two identical heavy chains. During early B cell development, immunoglobulins undergo rearrangement of various segments that lead to an extensive collection of immunoglobulin proteins. As this occurs, B cells simultaneously evolve from pro-B cells to pre-B cells to immature B cells via different extrinsic factors, such as interleukins and surface proteins, that vary by stage (i.e., CD19 for pro-B cells and CD79 for pre-B cells).

Immature B cells exit the bone marrow and migrate to secondary lymphoid organs such as the spleen and lymph nodes, where they undergo further maturation. In the white pulp of the spleen, signaling via the Notch pathway, B cell receptors (BCRs), and other mechanisms guides B cell differentiation through the “transitional” B cell stage into splenic marginal zone B cells or follicular B cells [67]. Marginal zone B cells secrete natural antibodies from their home in the spleen and help respond to blood-borne pathogens in T-cell-dependent and T-cell-independent fashions. Follicular B cells can be found in the spleen but will also circulate throughout the body, including lymph nodes and other tissues; these eventually serve as the main players in the T-cell-dependent B cell adaptive immune response and play a large role in immunological memory [66].

**Figure 2:**
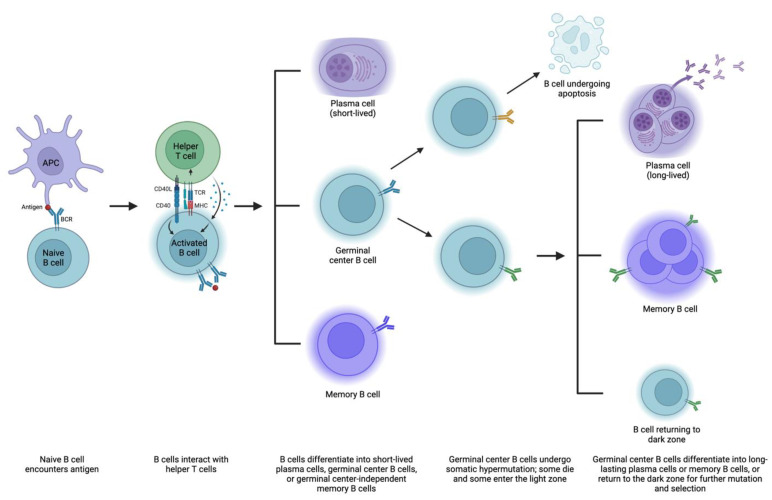
Mechanisms of B cell maturation and antibody formation. APC, antigen-presenting cell; BCR, B cell receptor; MHC, major histocompatibility complex; TCR, T cell receptor. Created with Biorender.

Immunological memory is due to both memory B cells and antibody-producing plasma cells [68]. These are formed in two main phases (Figure 2). First, a naïve B cell encounters an antigen (i.e., via a presentation from an APC), and the antigen binds to a BCR. This leads to downstream BCR signaling, internalization and processing of the antigen, and presentation of the antigen on MHC. Activated B cells then migrate to areas where they interact with helper T cells. From here, B cells can generally differentiate into one of three things: (1) plasma cells that produce low-affinity pathogen-specific antibodies and typically dissipate after an acute pathogenic insult, (2) germinal center B cells that will later become longer-lasting plasma cells via differentiation in the germinal center, and (3) germinal center-independent memory B cells. (Notably, most DSA production depends on T cells, but there may be some T-cell-independent processes, such as the formation of IgM antibodies and some ABO antibodies that can bypass T-cell-mediated steps [69,70].)

This second group of B cells goes through a second phase of memory acquisition. These B cells enter germinal centers within the follicle, starting in the dark zone, where they multiply and undergo somatic hypermutation, or a series of point mutations in heavy and light chain immunoglobulin sequences [68,71]. Some mutations are nonproductive and result in B cell apoptosis [72]. Surviving B cells then enter the light zone, with three potentially ensuing outcomes: (1) they can differentiate into long-lasting plasma cells that reside in the bone marrow and produce antibodies, (2) they can differentiate into memory B cells that occupy various tissues and remain on standby in case of antigen exposure, and (3) they can return to the dark zone and undergo additional somatic hypermutation and selection to refine antibody production [68].

Plasma cells are divided into short-lived and more durable cohorts, with the former transiently detected in the blood and the latter more stably housed in the bone marrow and lymphoid organs, among other organs [73]. Both can secrete highly specific antibodies in large amounts without requiring additional T cell help [74]. Memory B cells exposed to antigens can also become long-lasting plasma cells or re-enter germinal centers for somatic hypermutation, selection, and expansion. Isotype switching, whereby the B cell changes its class of antibody production (i.e., IgM to IgG), likely occurs predominantly in the light zone as well [75,76]. Memory B cells generated in the first phase of immunological memory (germinal center-independent) typically produce IgM, while those generated in the second phase often express other isotypes as they have undergone class switching [69]. IgM and IgG—as well as IgA, IgD, and IgE—have different capabilities, respond with different intensities to antigens, and survive for different lengths of time. Thus, there are a variety of routes by which B cells can become antibody-producing factories and contribute to the highly specialized adaptive immune response. Various mechanisms contribute to the fate of B cells, including BCR affinity for antigen, antigen structure, pathogen products, helper T cell activity, transcription factors, and other downstream signaling [68,77,78,79]. As with T cells, there is a subset of B cells (Bregs) tasked with suppressing certain autoimmune and alloimmune responses, but the mechanisms behind their development and differentiation are not well understood [80].

## 5. Epidemiology of T-Cell-Mediated Rejection and Antibody-Mediated Rejection

There are various types of allograft rejection classified by time course and mechanism. Regarding time course, rejection can be hyperacute, acute, or chronic, while the mechanism can be T-cell- or antibody-mediated.

Hyperacute rejection is antibody-mediated and is caused by preformed DSA, such as ABO and HLA antibodies [81]. It occurs shortly after vascular anastomoses are made during transplantation, and the diagnosis is typically made in the operating room. This is now quite rare due to pre-transplant crossmatch testing.

Acute rejection, which is defined as the acute worsening of allograft function with concomitant histopathological changes, can be T-cell- or antibody-mediated. Like hyperacute rejection, acute rejection has become much less common over time. This improvement has been attributed mostly to improved immunosuppressive medications, which mitigate T cell activity more than antibody production, hence a shifting focus from TCMR to ABMR over the past few decades [82]. The rate of acute rejection at one year for transplants performed in 2020 was 6–11%, compared to 30–40% in the 1990s and 10–20% in the early 2000s [83,84]. This rate is slightly lower in living-donor compared to deceased-donor kidney transplants.

Chronic allograft rejection is similarly defined by a decline in graft function and histopathological changes, but the decline in function is slow and progressive—typically more than one year after transplant—and the histopathological findings are distinct from those of acute rejection [81]. Chronic rejection still contributes greatly to graft failure despite advances in therapy, but this is difficult to quantify, as it can be subtle and the terminology surrounding it has evolved over the years. One large study found that 57% of subjects who developed new-onset late kidney allograft dysfunction were positive for DSA and/or C4d deposition on biopsy (a controversial diagnostic criterion in ABMR) and that these patients had an increased risk of subsequent graft failure compared to those without evidence of antibody-mediated injury [85]. Another study of protocol allograft biopsies in 315 patients found 56 kidney failures with 36 (64%) due to rejection (others included glomerulonephritis and polyoma virus nephropathy) [86]. Most of these cases were ABMR, probably ABMR, or mixed rejection rather than TCMR alone. Among rejection losses in this study, all had evidence of ABMR (including mixed rejection) by the time of failure, and TCMR alone did not cause allograft loss. This suggests that ABMR may play a larger role in chronic transplant rejection and allograft failure.

It is important to note the entity of subclinical rejection as well, which is when biopsy reveals histologic changes of acute rejection but graft function is stable [87]. Its incidence is also difficult to quantify, ranging from five to greater than 50% of allografts in the first three months post-transplant. However, it may be less common with current immunosuppressive regimens than it was in the past. Many studies have shown a relationship between subclinical rejection and allograft impairment, but not all, and subclinical ABMR is thought to portend worse outcomes compared to subclinical TCMR [87,88,89,90].

## 6. Pathophysiology of T-Cell-Mediated Rejection

Acute TCMR is defined by both interstitial inflammation and tubulitis, and/or by intimal arteritis, though arteritis can be seen in ABMR as well [91]. Chronic active TCMR is characterized by tubulitis, total cortical inflammation, and scarred cortical inflammation in the absence of other etiologies, or arterial intimal fibrosis with mononuclear inflammation and the formation of neointima. Each of these findings can be explained by the previously described mechanisms of T cell priming and differentiation.

In the context of kidney transplantation, recipient T cells circulate in the blood and peripheral lymphoid tissues. Either donor APCs with donor MHC, or donor-derived antigens on recipient APCs, are recognized by T cells in lymphoid tissues [92]. Depending on the class of MHC, either CD4 or CD8 on the T cell binds to MHC. Cell adhesion molecules and costimulatory signals help activate T cells and lead to clonal expansion and differentiation in response to the specific donor antigens to which they are exposed. T cells are then attracted to different parts of the allograft by different substances such as chemokines, which are made by cytokines from other inflammatory cells [93]. T cell migration and proinflammatory substances eventually result in the pathologic changes seen in TCMR and can lead to gross allograft dysfunction.

In tubulitis, for example, inflammatory cells produce interleukin-17 (IL-17) and tumor necrosis factor α, which leads to the production of chemokines such as CCL2, which draws T cells to the tubules, causing their accumulation and tubulitis. Tubular cells such as macrophages produce transforming growth factor β, which can cause tubular cells to undergo epithelial-mesenchymal transition and eventually infiltrate the interstitium and cause fibrosis. Endarteritis, which is seen in 20–40% of biopsies in acute rejection and is thought to be driven by T cells at least in part, features a distinct but similar series of interactions between T cells, antigen (this time on targeted endothelium), surface molecules, and inflammatory mediators [88,93,94]. This can progress to intimal fibrosis and thickening over time. These pathologic changes can result in signs and symptoms of rejection such as graft tenderness, anuria, edema, hypertension, increase in serum creatinine, proteinuria, and metabolic disturbances depending on the timeline and severity of the rejection episode [95,96].

## 7. Pathophysiology of Antibody-Mediated Rejection

The diagnosis of acute ABMR requires three criteria to be met: first, there must be histologic evidence of acute tissue injury, which includes microvascular inflammation, intimal or transmural arteritis, acute thrombotic microangiopathy, or acute tubular injury; second, there must be evidence of current or recent antibody interaction with the vascular endothelium, including linear C4d staining, microvascular inflammation, or increased expression of well-validated gene transcripts or classifiers associated with ABMR in the biopsy tissue; third, there must be serologic evidence of circulating DSA, though this can be substituted for C4d staining or validated gene transcripts or classifiers [91]. Chronic active ABMR has the same diagnostic criteria except that it requires evidence of chronic, rather than acute, tissue injury, which includes transplant glomerulopathy, severe peritubular capillary basement membrane multilayering, or new-onset arterial intimal fibrosis. On the other hand, chronic inactive ABMR has similar morphologic findings but no evidence of current or recent antibody interaction with the endothelium, and like prior documented active ABMR and/or DSA. As with TCMR, these criteria are rooted in the pathophysiology behind ABMR.

Roughly 25% of acute rejection episodes are at least partially due to antibodies against donor HLA, and other antigens ABO blood group antigens and endothelial cell surface antigens can also trigger antibody formation and ABMR [93,97]. Other antigens, such as minor histocompatibility antigens and self-proteins, have also been proposed as potential contributors to ABMR, though they play less of a role. In the case of the major antigenic targets HLA and ABO, naïve B cells in the follicle encounter HLA or ABO blood group antigens are presented by either donor or recipient APCs; they are activated via BCRs and co-receptors; other receptors are upregulated (such as CCR7) and downregulated (such as CXCR5), which causes B cells to migrate to helper T cells outside the follicle; the B cell internalizes the BCR-bound antigen and presents peptides on MHC Class II to CD4 T cells; and T helper cells then help determine the fate of these B cells [68]. B cells migrate to and from light and dark zones, where they undergo somatic hypermutation, cell division, sometimes apoptosis, and additional helper T-cell-mediated selection, which helps determine their fate—typically some form of memory B cell or plasma cell capable of producing antibodies. Long-lived plasma cells can then produce antibodies from the bone marrow in perpetuity without T cell help [97]. As previously mentioned, some IgM and ABO antibodies may be formed in limited cases in a T-cell-independent fashion.

While ABMR is mediated by antibodies, however, antibodies do not directly kill allograft cells. Rather, allograft destruction occurs via the activation of the complement system and the actions of other cytotoxic cells [98]. Complement can be activated via three pathways: the classical pathway, the lectin pathway, and the alternative pathway [99]. The classical pathway is the most relevant in ABMR, as this pathway is triggered by antibodies. This pathway starts when an antibody-antigen complex on a cell binds the C1 complex (made up of complement proteins C1q, C1r, and C1s) and ends with cell lysis [100]. In the context of allograft rejection, IgG or IgM bound to antigens on graft endothelium binds to the C1 complex to initiate the complement cascade; the subsequent steps in the process are outlined here but described more thoroughly by Colvin and Smith in their 2005 review [97]. After this initial binding occurs, C1r is cleaved, which activates C1s, which activates C2 and C4. C4 is cleaved into C4a and C4b, which is inactivated to C4d by factor I. C4d then remains bound to tissue, hence its place in the diagnostic criteria for ABMR [101]. C4b then combines with C2a to form C4b2a, the classical pathway C3 convertase; C3 convertases are also produced by the lectin and alternative pathways. C3 is cleaved to C3a and C3b by these convertases, with subsequent formation of membrane attack complex (MAC; C5b, C6-9). The MAC, thereafter, causes cell lysis of the allograft cells.

Certain complement cleavage products, such as C3a and C5a, also cause damage by attracting neutrophils, macrophages, and mast cells to the allograft, causing inflammation. These and other products of the complement cascade can also cause conformational changes in endothelial cells that lead to the production of proinflammatory molecules, such as cytokines, adhesion molecules, and growth factors. These growth factors have varying downstream effects on the allograft: for example, the basic fibroblast growth factor can cause the proliferation of endothelial cells, and the tissue factor can cause thrombotic injury resembling thrombotic microangiography in the graft [97,102,103].

Finally, there are some mechanisms by which antibodies can interact with endothelial cells independently of complement [97]. For example, antibodies may be able to activate and cause the proliferation of endothelial cells via intracellular signaling, phosphorylation, and chemokine production. The antibody can also lyse cells via antibody-dependent cell-mediated cytotoxicity, which is mediated by natural killer cells and macrophages [97]—more precisely, the Fc receptor on these effector cells to cross-links with target cells, which triggers signaling pathways in the effector cells that cause cytotoxic granule release and kills target cells via perforin and granzyme [104]. However, it is somewhat unclear as to how and to what extent these complement-independent processes contribute to rejection. On the other hand, the injury mediated by the complement cascade results in the same signs and symptoms as TCMR, and ABMR and TCMR must be distinguished via biopsy.

## 8. Limitations to Current Definitions of Rejection

The criteria outlined here for defining TCMR and ABMR are defined by the international Banff classification, which has been the gold standard for the diagnosis of kidney transplant rejection since 1993 and is evaluated and updated regularly [105]. However, this system has many complexities and limitations. C4d was once thought to be a distinct marker for ABMR, but it was found that C4d can be present without morphologic evidence of rejection, and it can be absent in ABMR [106]. The meaning of this is unclear, with one study showing that patients with C4d-positive biopsies without evidence of rejection had a higher risk of developing ABMR [107]. Furthermore, there is an entity of ABMR in which DSA is not present [108]. This is why the Banff classification now includes an alternative to C4d positivity: “increased expression of gene transcripts/classifiers strongly associated with ABMR” [91,105].

The concept of DSA-negative ABMR has changed the transplant landscape. Many studies have shown that patients with molecular findings of ABMR often have no evidence of circulating DSA, with a frequency as high as 66% in one study [109], across different detection methods, MFI cutoff values, and definitions for things such as microvascular inflammation [57]. This likely has prognostic implications. In one study, HLA-DSA-negative ABMR was associated with less histologic persistence at follow up, and HLA-DSA-negative AMBR has been associated with better outcomes than HLA-DSA-positive ABMR in some studies and similar outcomes in others [108,110,111]. Some potential mechanisms of HLA-DSA-negative ABMR include the concept of “missing self,” in which recipient natural killer cells sense the absence of self-HLA class I on allograft endothelial cells, rather than DSA binding to the allograft [112]; genetic mismatch of non-HLA haplotypes [113]; and innate allorecognition of non-HLA antigens by monocytes and macrophages [57].

Rejection based on Banff criteria is defined quantitatively by examining cortical tubules and interstitium histologically. However, some of these definitions are poorly defined, such as the number of inflammatory cells required to diagnose glomerulitis, or subjective, such as the criterion of “endothelial enlargement” [105]. Certain borderline findings raise “suspicion” for rejection but make it difficult to diagnose with authority. Additionally, there are some histologic findings that do not inherently identify whether ABMR or TCMR, or a separate injury unrelated to rejection, was the cause. Studies have demonstrated discordance between the reference standard and clinicians’ diagnoses using the Banff classification, which may have therapeutic implications [114]. A recent article reported the development of an automated histological classification system that was associated with less misdiagnosis of allograft rejection and improved risk stratification of long-term transplant outcomes, highlighting the role of automation and artificial intelligence in diagnosing rejection [115].

## 9. Outcomes and Advances in Understanding of Pathophysiology of Rejection

Some of the greatest milestones in the understanding of the pathophysiology of rejection have included the discovery of HLA and DSA, the invention of crossmatch testing, the development of pharmacotherapy to target pathways of rejection, investigation into the role of regulatory cells, and increased research on autoreactivity and the role of self.

As outlined above, HLA was not discovered until years after the first kidney transplant was performed, and its role in transplantation was identified still later [23,41]. When the role of HLA typing was established, it initially focused on *HLA-A*, *HLA-B*, and *HLA-DR*, and sometimes *HLA-DQ*, mostly because they are thought to have the biggest immunologic impact in transplant, but in part due to limitations in typing techniques. Now that more extensive HLA typing is performed regularly, more research is being conducted on the impact of mismatches for other antigens, such as HLA-C and HLA-DP, with inconclusive but intriguing results that necessitate more research [116,117,118]. With improvements in typing methodology, understanding the impact of mismatches at loci beyond the traditionally implicated antigens could theoretically improve donor-recipient matching in the future.

Despite these improvements, there is still uncertainty as to the level of depth that should be pursued with HLA typing: examining antigen mismatches at three loci seems far too simple, but analyzing thousands upon thousands of alleles is not yet practical. Emerging alternative strategies such as eplet matching help to bridge this gap and may represent an improved approach to assessing recipient risk of rejection [119,120]. This is founded on an understanding of epitopes and eplets. Epitopes are portions of antigens that interact with a particular antibody, and an eplet is a functional unit of an epitope that is a fraction of the size but can determine antibody specificity. Researchers have defined a list of such eplets that may act in the alloimmune response in transplant, though further investigation is needed to determine their biology and relevance [119,121]. Nonetheless, recent studies have demonstrated the early benefits of an eplet matching approach. For example, Kishikawa et al. found that class II eplet mismatch was a risk factor for dsDNA and ABMR in transplant recipients and had even greater significance as a risk factor than the number of traditional HLA class II mismatches [120].

DSA is a related area that has similarly seen recent improvements. DSA can be detected via CDC and flow cytometry crossmatch, and more recently solid-phase methods have been developed that can identify anti-HLA specificities of DSA in addition to simply detecting DSA [23,25,26,27,28]. Furthermore, research has demonstrated that patients may have an increased risk of ABMR with DSA detected using these more sensitive, newer methods even with negative CDC and flow cytometry crossmatch [26]. More studies are needed to evaluate this risk more fully and determine an approach to immunosuppression in these patients.

The development and evolution of immunosuppressive medications have dramatically impacted rates of rejection and overall outcomes. While these improvements are due largely to the understanding of the pathophysiology of rejection, a complete discussion of immunosuppression is outside of the scope of this review. One relevant avenue for therapeutic intervention that is particularly relevant, however, involves harnessing the immune tolerance powers of regulatory cells. Tregs in particular are becoming increasingly recognized for their role in minimizing the unnecessary immune responses to self or transplant that lead to transplant rejection [64]. These cells migrate to sites of inflammation and suppress effector T cells and other immune responses, both innate and adaptive, that mediate rejection. There is a general understanding that the balance of Tregs and effector T cells can determine whether a transplant will be rejected; a balance grossly in favor of Tregs could perhaps lead to tolerance of the transplant. Injection of Tregs has demonstrated safety and potential as a therapeutic intervention in kidney transplant recipients, opening the door to further investigation of regulatory cells in transplant [122].

Another emerging area in transplant rejection is the concept of rejection outside of the clear-cut boundaries of ABMR and TCMR; this implicates other processes underlying rejection, such as autoreactivity against self-antigens and innate allorecognition mediated by natural killer cells and monocytes [57]. Improved understanding of the pathophysiology of rejection, in conjunction with research on agents targeting known and more up-and-coming pathways, should lead to continued improvements in the prevention and treatment of rejection in the future.

## 10. Conclusions

Kidney allograft rejection continues to be a major cause of allograft loss. Understanding the risk factors that predispose to rejection, the mechanisms behind T cell priming and antibody formation, the epidemiology of rejection, and the pathophysiology of ABMR and TCMR has led to improved outcomes and is crucial for continued progress. The next frontier in this sphere is the development of better techniques of immune monitoring, immunosuppression with minimal adverse effects, and enhanced elucidation of eplet matching.

## Data Availability

Not applicable.
